# PIM1 drives lipid droplet accumulation to promote proliferation and survival in prostate cancer

**DOI:** 10.1038/s41388-023-02914-0

**Published:** 2023-12-14

**Authors:** Shailender S. Chauhan, Andrea L. Casillas, Andres D. Vizzerra, Hope Liou, Amber N. Clements, Caitlyn E. Flores, Christopher T. Prevost, David F. Kashatus, Ashley J. Snider, Justin M. Snider, Noel A. Warfel

**Affiliations:** 1 Department of Cellular and Molecular Medicine, University of Arizona, Tucson, AZ 85724 USA; 2 Cancer Biology Graduate Program, University of Arizona, Tucson, AZ 85721 USA; 3 Department of Microbiology, Immunology and Cancer Biology, University of Virginia Health System, Charlottesville, VA 22908 USA; 4 Department of Nutritional Sciences, College of Agriculture and Life Sciences, University of Arizona, Tucson, AZ 85721 USA; 5 University of Arizona Cancer Center, University of Arizona, Tucson, AZ 85724 USA

**Keywords:** Prostate cancer, Lipid signalling

## Abstract

Lipid droplets (LDs) are dynamic organelles with a neutral lipid core surrounded by a phospholipid monolayer. Solid tumors exhibit LD accumulation, and it is believed that LDs promote cell survival by providing an energy source during energy deprivation. However, the precise mechanisms controlling LD accumulation and utilization in prostate cancer are not well known. Here, we show peroxisome proliferator-activated receptor α (PPARα) acts downstream of PIM1 kinase to accelerate LD accumulation and promote cell proliferation in prostate cancer. Mechanistically, PIM1 inactivates glycogen synthase kinase 3 beta (GSK3β) via serine 9 phosphorylation. GSK3β inhibition stabilizes PPARα and enhances the transcription of genes linked to peroxisomal biogenesis (PEX3 and PEX5) and LD growth (Tip47). The effects of PIM1 on LD accumulation are abrogated with GW6471, a specific inhibitor for PPARα. Notably, LD accumulation downstream of PIM1 provides a significant survival advantage for prostate cancer cells during nutrient stress, such as glucose depletion. Inhibiting PIM reduces LD accumulation in vivo alongside slow tumor growth and proliferation. Furthermore, TKO mice, lacking PIM isoforms, exhibit suppression in circulating triglycerides. Overall, our findings establish PIM1 as an important regulator of LD accumulation through GSK3β-PPARα signaling axis to promote cell proliferation and survival during nutrient stress.

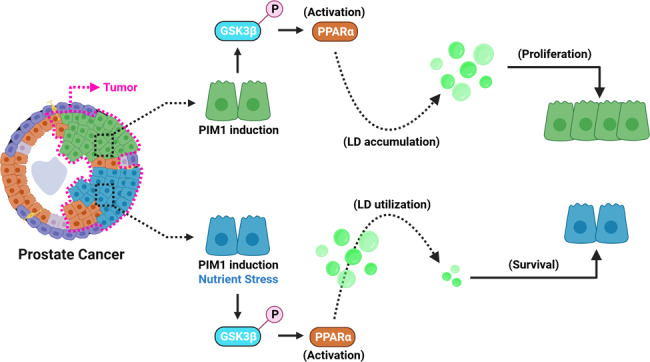

## Introduction

Lipid droplets (LDs), also known as lipid bodies, are composed of a neutral lipid core surrounded by a phospholipid monolayer, and they are localized primarily to the cytoplasm [1] or nucleus [2] depending on cell type. LDs emerge from the endoplasmic reticulum through a regulated process involving nucleation, growth, budding, and detachment [1, 3]. Functionally, these dynamic organelles are positioned at the center of lipid storage and energy homeostasis [4]. In addition, the accumulation of LDs, especially in non-adipocytes has been associated with various pathologic conditions [5]. More recently, numerous investigations have reported that transformed cells maintain elevated levels of intracellular LDs via alteration in the balance between lipids generation (uptake, synthesis, and remodeling) and lipolysis [6]. Accruing evidence has established a connection between lipolysis or lipogenesis and tumor growth in prostate cancer [7–10], suggesting that targeting the utilization of LD-associated lipids could be an effective strategy for cancer therapy. As evidence of this, a key regulator of lipid homeostasis, SREBPs (Sterol Regulatory Element-Binding Proteins), and a crucial sensor for nutrient signaling, the mammalian target of rapamycin (mTOR), have been reported to influence lipogenesis to drive prostate cancer growth and progression [7–9].

Restricted nutrient availability in the tumor microenvironment due to varying degrees of vascularization [11] leads to nutrient stress. Overcoming nutrient stress is essential for tumor growth and progression. When nutrients are limited, cancer cells must rely on alternative energy sources due to a lack of glucose. One of the mechanisms cancer cells adopt to maintain growth or survival under nutrient stress is the utilization of lipids that are commonly sequestered in LDs [12–14]. LDs store neutral lipids (triglycerides (TAs) and Cholesterol ester (CE)) that can be shuttled into the mitochondria to promote ATP production [14, 15]. High-grade and metastatic prostate cancer exhibit an uncharacteristic accumulation of CE in LDs [10], which promotes tumor growth [16]. Under restricted or low access to glucose, prostate cancer predominantly relies on fatty acid oxidation for growth and proliferation [17, 18]. Therefore, identifying the signaling pathways that control LD accumulation and their utilization is important for understanding the progression and improving the treatment of prostate cancer.

The *Proviral Integration site* for Moloney murine leukemia virus 1 (PIM1) kinase is a member or a family of oncogenic Ser/Thr kinases whose levels are elevated in prostate cancer and have proven to promote tumor proliferation and resistance to therapy [19]. PIM1 promotes tumor progression through various mechanisms, impacting cell cycle progression, proliferation, and survival [20]. As a result, PIM is a promising anti-cancer therapeutic target and several small-molecule pan-PIM inhibitors have shown efficacy in phase I/II clinical trials [21]. Previous studies have implicated PIM as a regulator of cellular energy metabolism through its positive effect on mitochondrial biogenesis, glycolysis, and adipogenesis [22, 23]. However, the connection between alterations in lipid metabolism or LD accumulation and PIM kinases has never been investigated in cancer cells.

Here we identify PIM1 as a driver of LD accumulation and demonstrate a critical role for this event for prostate cancer cell proliferation and survival during nutrient stress. Overexpression of PIM1 increases LD number and size in both in vitro and in vivo models of prostate cancer. We identify a novel signaling axis whereby upregulation of PIM1 inhibits GSK3β through direct phosphorylation at serine 9 (S9). Inhibition of GSK3β by PIM1 results in increased PPARα stability and nuclear expression, which favors enhanced peroxisomal biogenesis to complement LD accumulation. The accumulation of LDs downstream of PIM1 enhances prostate cancer survival during nutrient stress. Finally, targeting PIM1 or PPARα signaling was sufficient to abrogate the proliferative and survival functions associated with LD accumulation or PIM1 in prostate cancer.

## Results

### PIM1 kinase is essential for LD accumulation in prostate cancer

**Fig. 1:**
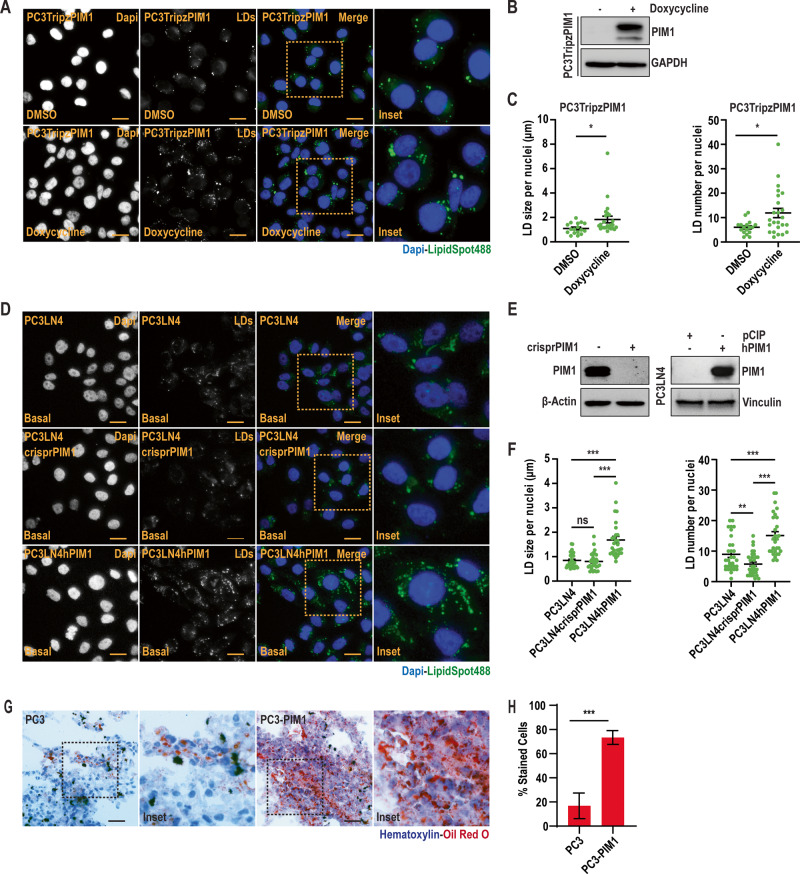
PIM1 kinase is essential for LD accumulation in prostate cancer. **A** Representative images of LDs in PC3TripzPIM1 cells following doxycycline treatment (50 ng/ml, 24 h). **B** Western blot of PC3TripzPIM1 cells following doxycycline treatment (50 ng/ml, 24 h). **C** Quantification of LD size and number per nuclei (*n* > 30 cells/group). **D** Representative images of basal levels of LDs, and (**E**) western blotting for PIM1 expression in the indicated cell lines. **F** Quantification of LD size and number per nuclei (*n* > 30 cells/group). **G** Representative images of LDs in tumor sections from PC3 and PC3-PIM1 xenograft tumors; LDs are in red (Oil Red O) and nuclei in blue (Hematoxylin). **H** Quantification of oil red o staining as % stained cells. At least 30 cells from 6 random fields were analyzed per treatment group. *n* = 3, mean ± SEM. ***p ≤ 0.001. For staining in (**A**) and (**D**), LDs in green (LipidSpot488) and nuclei in blue (Dapi). Scale bars, 50 µm. *n* = 3, mean ± SEM. *p ≤ 0.05, **p ≤ 0.01, ***p ≤ 0.001.

Recent investigations have described LDs as a hallmark of cancer [6], and the enzymes involved in their biogenesis/processing positively regulate prostate cancer cell growth [16]. Because PIM kinases are associated with tumor aggressiveness across a wide spectrum of solid tumors, we speculated that PIM1 could influence LD accumulation in prostate cancer. To test this, we utilized a prostate cancer cell line (PC3TripzPIM1) stably expressing a doxycycline-inducible PIM1 vector (TripzPIM1) (Fig. S1A, B). To rule out any non-specific effects of doxycycline treatment on PIM isoforms at a dosage of 50 ng/ml, we assessed PIM1 levels in PC3 cells expressing doxycycline-inducible empty vector (TripzEV) and TripzPIM1 (Fig. S1B). Then, PC3TripzPIM1 cells were treated with doxycycline (50 ng/ml, 24 h) to induce PIM1, and LD accumulation was assessed by staining for LipidSpot488 (Fig. 1A, B). Quantitative analysis revealed a greater than two-fold increase in the LD number per nuclei in cells overexpressing PIM1 compared to control (Fig. 1C).

To further validate these effects on LDs, we generated PC3LN4 prostate cancer cells lacking PIM1 (PC3LN4crisprPIM1) or stably overexpressing PIM1 (PC3LN4hPIM1) (Fig. 1E). Consistent with our findings in PC3 cells, PIM1 upregulation significantly increased LD accumulation (Figs. 1D, S1C), showing increased average LD size and number per nuclei (Figs. 1D, F, S1C, D). Interestingly, loss of PIM1 decreased LD number per nuclei but did not affect LD size (Fig. 1D, F). The discrepancy in LD size with PIM1 loss could be attributed to the compensatory effect of PIM kinase isoforms (PIM2, PIM3). To test the role of PIM1 isoform in LD accumulation, we performed LipidSpot488 staining of wild-type mouse embryonic fibroblasts (WT MEFs), triple knockout (TKO; lacking PIM1, PIM2, and PIM3), TKO-PIM1 (stable addback of PIM1 only), (Fig. S1E, F). Qualitative and quantitative analyses revealed a significant decrease in LD size and number per nuclei in TKO MEFs compared to WT MEFs, and LD number was restored to basal levels in MEFs expressing PIM1 (Fig. S1G). We then questioned if PTEN or androgen receptor (AR) status can affect LD accumulation associated with PIM1 in prostate cancer. To this end, we used DU145 (PTEN positive) cells stably overexpressing PIM1 (hPIM1) or backbone (pCIP). We observed a similar, significant increase in LD accumulation and size or number per nuclei (Fig. S1H, I). Similar results were also observed using 22Rv1 cells with PTEN stably knocked out, indicating that PIM controls LD accumulation independent of PTEN status (Fig S1L−P). To test whether AR status impacts this process, LNCaP cells (AR positive) were treated PIM447 and LDs were quantified. PIM inhibition significantly reduced LD in LNCaP to a similar extent as cells lacking functional AR (S1J, K). Thus, the effects of PIM1 on LD in prostate cancer are independent of AR or PTEN. Finally, we investigated whether the effects of PIM1 on LD accumulation are replicated in vivo using a xenograft model of prostate cancer. Tumors isolated from mice injected with PIM1 overexpressing cells (PC3-PIM1) showed an approximately four-fold increase in Oil Red O positive cells as compared to control tumors (PC3) (Fig. 1G). Altogether, these data demonstrate that PIM1 promotes LD accumulation in prostate cancer.

### PIM1 kinase inhibits GSK3β to induce LD accumulation

**Fig. 2:**
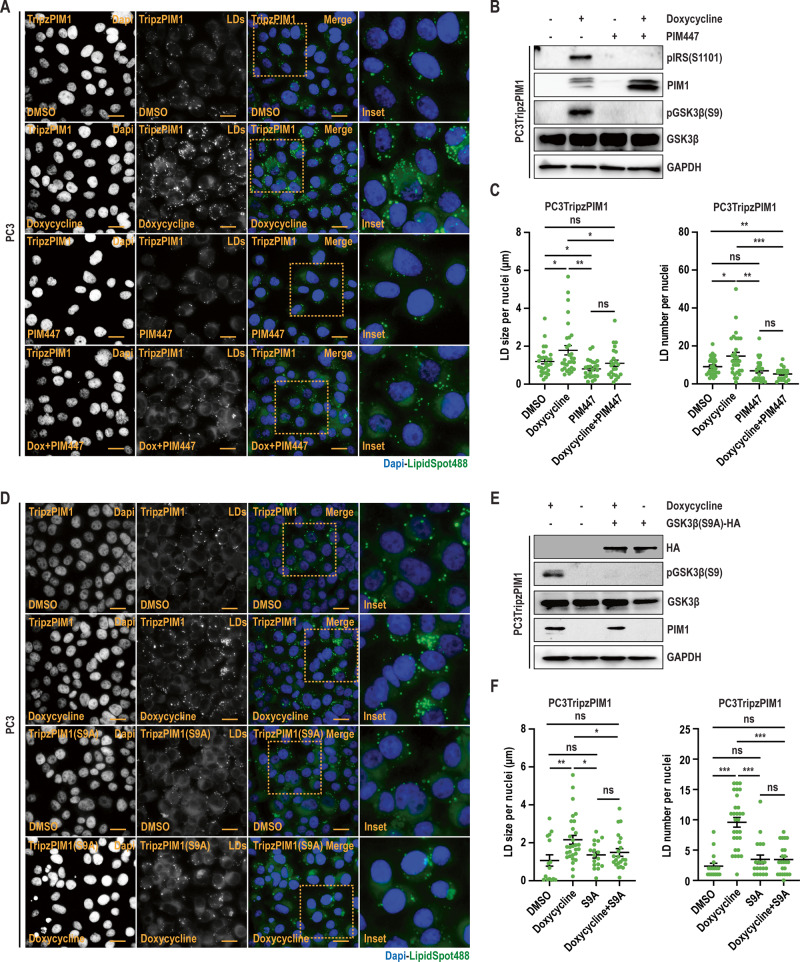
PIM1 induces LD accumulation through GSK3β inhibition. **A** Representative images of LDs in PC3TripzPIM1 cells following doxycycline treatment (50 ng/ml, 24 h) alone, and in combination with PIM inhibition (PIM447, 3 µM, 24 h). **B** Western blotting of PC3Tripz-PIM1 cells following doxycycline treatment (50 ng/ml, 24 h) alone, and in combination with PIM inhibition (PIM447, 3 µM, 24 h). **C** Quantification of LD size and number per nuclei (*n* > 30 cells/group). **D** Representative images of LDs and (**E**) western blotting of PC3TripzPIM1 cells following doxycycline treatment (50 ng/ml, 24 h) expressing vector or GSK3β (S9A) mutant. **F** Quantification of LD size and number per nuclei (*n* > 30 cells/group). For staining in (**A**) and (**D**), LDs in green (LipidSpot488) and nuclei in blue (Dapi). Scale bars, 50 µm. *n* = 3, mean ± SEM. *p ≤ 0.05, **p ≤ 0.01, ***p ≤ 0.001.

Recent studies have liked the inhibition of GSK3β to LD accumulation [24]. The activation state of GSK3β is canonically dictated by inhibitory phosphorylation at S9. Thus, we investigated whether PIM1 altered GSK3β phosphorylation and activity. First, we assessed S9 phosphorylation in various cancer models (prostate, colon, renal, and lung) after overexpression of PIM1. In all cell lines, upregulation of PIM1 significantly increased GSK3β (S9) phosphorylation (Fig. S2A). To confirm that increased S9 phosphorylation was specific to PIM1, we treated PC3TripzPIM1 cells with doxycycline (50 ng/ml, 24 h) or PIM kinase inhibitor (PIM447, 3 µM, 24 h) alone and in combination. The inactivation of PIM1 was assessed by pIRS (S1101) status, which is a direct target of PIM1 [25]. Overexpression of PIM1 increased pGSK3β (S9) phosphorylation, whereas no S9 phosphorylation was detected after treatment with PIM447, regardless of PIM1 overexpression (Fig. 2B). As expected, increased S9 phosphorylation by PIM1 tracked with increased LD size and number per nuclei (Fig. 2A−C). Next, we tested if direct inhibition of GSK3β using a specific inhibitor (CHIR) could mimic the effects of PIM1 induction on LD accumulation. PC3 prostate cancer cells were treated with CHIR (50 nM, 24 h) and stained for LDs using LipidSpot610. Western blot analysis was performed to confirm GSK3β inhibition, as determined by increased levels of β-catenin or cyclin D1 following CHIR treatment (Fig. S2C). As speculated, inhibiting GSK3β increased LD size and number per nuclei (Fig. S2B−D) to a similar extent as PIM1 induction. Next, we asked whether inhibition of GSK3β by PIM1 is necessary for PIM1 to regulate LD accumulation. To this end, we expressed a GSK3β(S9A)-HA plasmid with S9 mutated to alanine (S9A) in PC3TripzPIM1 cells, followed by PIM1 induction (doxycycline, 50 ng/ml, 24 h). Western blot analysis showed that S9A mutation was sufficient to block the inhibitory effect of PIM1 on GSK3β (Fig. 2E). Importantly, expression of an S9 mutant of GSK3 compromises the ability of PIM1 to increase LD size and number per nuclei (Fig. 2D−F). Collectively, the results indicate that PIM1 regulates LD accumulation in prostate cancer by inhibitory phosphorylation of GSK3β at S9.

### GSK3β inhibition enhances peroxisomal biogenesis through PPARα to induce LD accumulation

**Fig. 3:**
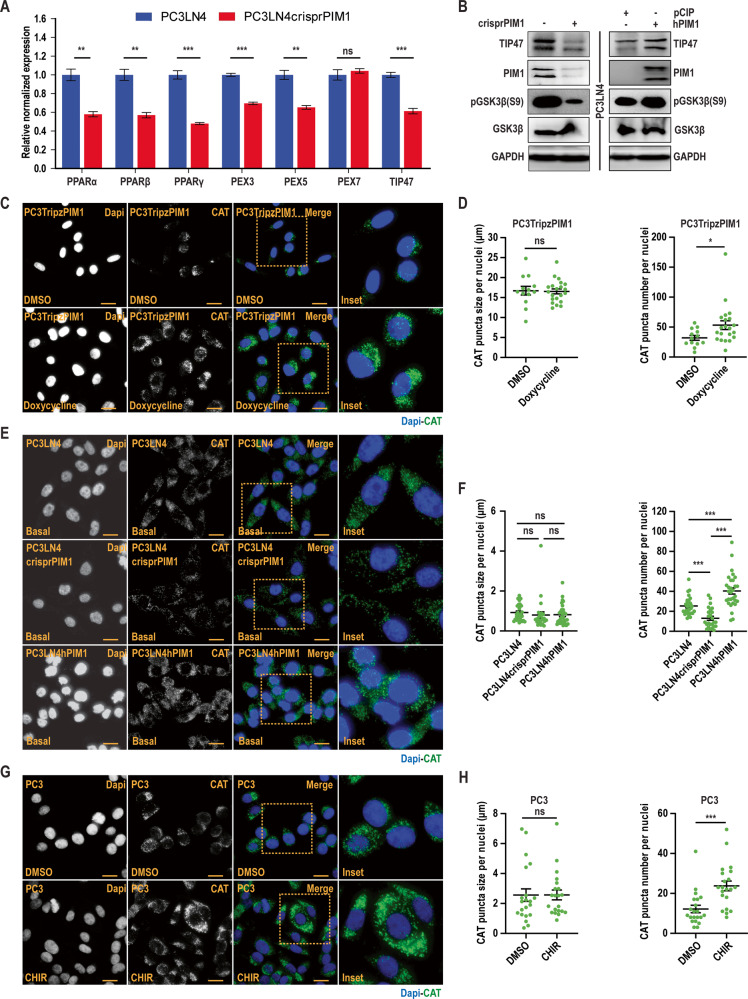
GSK3β inhibition enhances peroxisomal biogenesis through PPARα to induce LD accumulation. **A** Relative normalized expression of indicated genes relating to PPAR signaling (PPARα, β, γ), peroxisomal biogenesis (PEX3, PEX5, and PEX7), and Lipid droplet growth (TIP47) in PC3LN4 and PC3LN4crisprPIM1 cells. **B** Western blotting of TIP47 expression and GSK3β S9 phosphorylation in the indicated cell lines. **C** Representative images of peroxisomal marker catalase (CAT) in PC3TripzPIM1 cells following doxycycline treatment (50 ng/ml, 24 h). **D** Quantification of CAT puncta size and number per nuclei (*n* > 30 cells/group). **E** Representative images of basal CAT staining in the indicated cell lines. **F** Quantification of CAT puncta size and number per nuclei (*n* > 30 cells/group). **G** Representative images of CAT in PC3 cells following CHIR treatment (50 nM, 24 h). **H** Quantification of CAT puncta size and number per nuclei (*n* > 30 cells/group). For (**C**), (**E**), and (**G**), CAT is in green, and nuclei in blue (Dapi). Scale bars, 50 µm. *n* = 3, mean ± SEM. *p ≤ 0.05, ***p ≤ 0.001.

Peroxisome proliferator-activated receptor alpha (PPARα) is a ligand-activated transcription factor that controls the levels of triglycerides (TGs), a major component of LD core. Previous work indicates that GSK3β can regulate PPARα stability [26, 27] through proteasomal degradation [28]. Thus, we hypothesized that PIM1 induction could indirectly activate PPARα signaling to facilitate LD accumulation. First, we examined PPARα stability using a cycloheximide chase assay in DU145 prostate cancer cells stably expressing vector (DU145pCIP) or PIM1 (DU145hPIM1). PPARα degradation was significantly slower in cells overexpressing PIM1 compared to controls (Fig. S3A). PPARs act in the nucleus, which compelled us to speculate that the increase in protein stability following PIM1 induction increases the nuclear accumulation of PPARα. Immunofluorescence analysis of cells co-stained for PPARα (red) and DAPI (blue) showed roughly a two-fold increase in PPARα nuclear fluorescence in cells overexpressing PIM1 (Fig. S3B, C). We next checked the relative mRNA expression of genes related to PPAR signaling (PPARα, β, and γ), peroxisomal biogenesis factor proteins (PEX3, 5, and 7), and LD growth (TIP47) at basal level in PC3LN4 or PC3LN4crisprPIM1 cells. Loss of PIM1 significantly decreased the expression of genes from each category (Fig. 3A). We validated these results along with the phosphorylation state of GSK3β(S9) in the previously described PC3LN4 cells overexpressing or lacking PIM1. There was a strong correlation between PIM1 expression, GSK3β inactivation, and Tip47 levels (Fig. 3A, B). To examine the effects of PPARα on peroxisomal biogenesis, we stained for a specific marker of peroxisomes (catalase, CAT) in PC3 cells overexpressing PIM1 or treated with CHIR to directly inhibit GSK3β. Catalase staining significantly increased following GSK3β inhibition, irrespective of whether it was due to PIM1 or chemical inhibition (Fig. 3C, E, G). Interestingly, quantitative analysis revealed a significant increase in peroxisome number but not size per nuclei (Fig. 3D, F, H). To show that GSK3β is downstream of PIM1 in the context of LD accumulation, PC3 cells were treated with CHIR or PIM447, alone or in combination, and stained for CAT (Fig. S4A) or LDs (Fig. S4C). While individual treatments showed increased CAT and LD with GSK3β inhibition and decreased CAT and LD with PIM inhibition (Figs. 1–3), the combination had a similar effect as CHIR alone on both catalase and LD (Fig. S4A–D). Taken together, these data indicate that PIM1 amplifies peroxisomal biogenesis by inhibiting GSK3β-PPARα signaling to induce LD accumulation in prostate cancer.

### PPARα inhibition abrogates the effect of PIM1 kinase on LD accumulation and proliferation

**Fig. 4:**
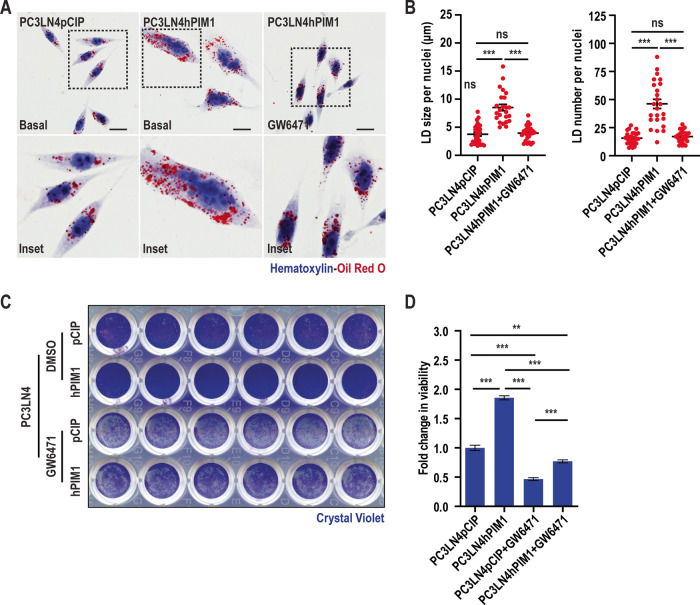
PPARα inhibition abrogates the effect of PIM1 LD accumulation and prostate cancer proliferation. **A** Representative images of LDs in PC3LN4pCIP (basal) and PC3LN4hPIM1 (basal and with PPARα inhibitor, GW6471 treatment, 4 µM, 24 h) cells. LDs are in red (Oil Red O) and nuclei in blue (Hematoxylin). Scale bars, 50 µm. **B** Quantification of LD size and number per nuclei (*n* > 27 cells/group). *n* = 3, mean ± SEM. ***p ≤ 0.001. **C** Representative images of crystal violet staining of PC3LN4pCIP and PC3LN4hPIM1 cells treated with DMSO or PPARα inhibitor (GW6471, 4 µM, 72 h). **D** Quantification of crystal violet staining representing fold change in cell viability. *n* = 3, mean ± SEM. **p ≤ 0.01, ***p ≤ 0.001.

LD accumulation and utilization are closely linked to energy homeostasis and are considered essential for tumor proliferation [6]. Thus, we designed experiments to test the importance of PIM1-induced LD accumulation for prostate cancer cell proliferation. Given that LD accumulation in our experimental setting occurred through the GSK3β-PPARα signaling axis downstream of PIM1, we hypothesized that blocking PPARα could counteract the known positive effect of PIM1 induction on cell proliferation in prostate cancer. To this end, we treated PC3-LN4 cells with a specific inhibitor for PPARα (GW6471, 4 µM, 24 h), and cells were stained for LDs using Oil O Red. As expected, GW5471 significantly reduced both the size and number of LDs induced by PIM1 overexpression (Fig. 4A, B). We next addressed the impact of altering LDs on proliferation using crystal violet staining to quantify cell number. PIM1 overexpression (PC3LN4hPIM1) significantly increased proliferation compared to controls. Interestingly, GW6471 treatment reduced cell number below that of parental cells (PC3LN4pCIP), even in cells overexpressing PIM1 (Fig. 4C, D). However, PC3LN4hPIM1 maintained higher proliferation as compared to PC3LN4pCIP in the presence of GW6471, supporting the involvement of PIM1 in regulating cell proliferation independent of PPARα signaling axis. Therefore, it is possible that LD accumulation could be one of the earlier events dictating the proliferative functions of PIM1 mediated through PPARα signaling. Collectively, these data indicate that activation of PPARα downstream of PIM1 is essential for LD accumulation and underscore the role of LD accumulation in mediating PIM1-associated cell proliferation in prostate cancer.

### PIM1 kinase alters lipid composition in prostate cancer

**Fig. 5:**
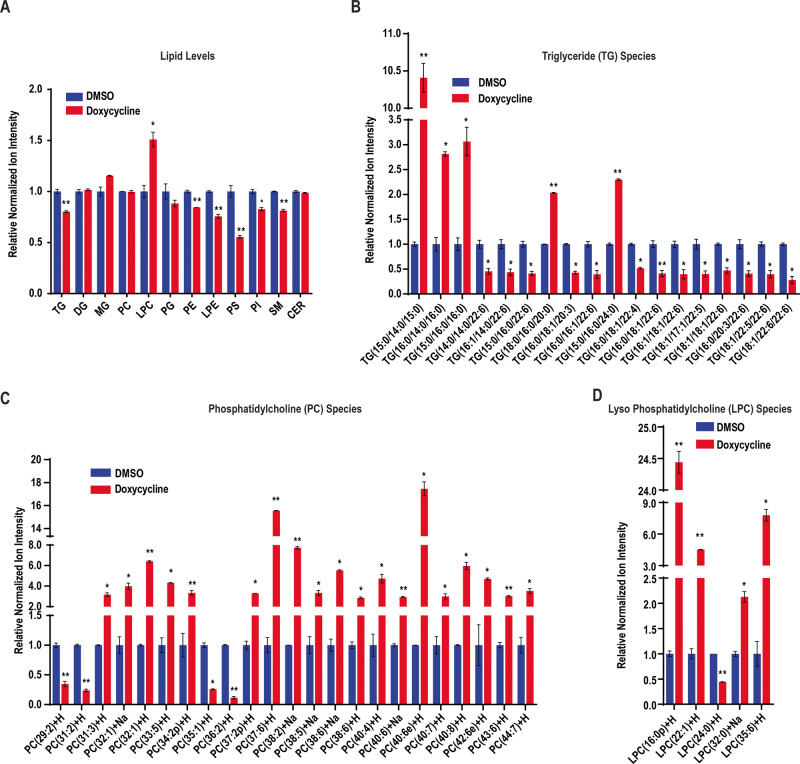
Analysis of lipid composition with PIM1 induction in prostate cancer. PC3Tripz-PIM1 cells were treated with DMSO or doxycycline (50 ng/ml, 24 h) and lipids were extracted and analyzed by liquid chromatography-tandem mass spectrometry (LC-MS/MS). **A** Levels of various lipids in PC3Tripz-PIM1 cells. Raw values were normalized, and fold change vs DMSO was calculated to generate relative normalized ion intensity values. *n* = 3, mean ± SEM. *p ≤ 0.05, **p ≤ 0.01. **B** Relative normalized ion intensity values for most abundant TG species detected. Raw values were normalized, and fold change vs DMSO was calculated to generate relative normalized ion intensity values. *n* = 3, mean ± SEM. *p ≤ 0.05, **p ≤ 0.01. **C** Relative normalized ion intensity values for most abundant PC species detected. Raw values were normalized, and fold change as vs DMSO was calculated to generate relative normalized ion intensity values. *n* = 3, mean ± SEM. *p ≤ 0.05, **p ≤ 0.01. **D** Relative normalized ion intensity values for most abundant LPC species detected. Raw values were normalized, and fold change as vs DMSO was calculated to generate relative normalized ion intensity values. *n* = 3, mean ± SEM. *p ≤ 0.05, **p ≤ 0.01.

The distinct composition of LDs, comprising a neutral lipid core surrounded by a phospholipid monolayer, makes them a suitable site for storing lipids as energy reservoirs [4]. In cancer cells, lipid composition is desirably altered through metabolic reprogramming that increases production, storage, and uptake of lipids to favor survival and proliferation [29]. Since LDs primarily store neutral lipids in the form of triglycerides (TG) and sterol ester (SE), we sought to confirm if increased LD accumulation following PIM1 upregulation is reflected at the level of different lipid species. Therefore, we treated PC3TripzPIM1 cells with DMSO or doxycycline (50 ng/ml, 24 h) and performed an untargeted lipidomic analysis involving liquid chromatography-mass spectrometry (LC-MS) to assess lipid composition. Contrary to our expectations, Total TG levels decreased, whereas other species (phosphatidylethanolamine (PE), Lyso-phosphatidylethanolamine (LPE), phosphatidylserine (PS), phosphatidylinositol (PI), and sphingomyelin (SM)), lyso-phosphatidylcholine (LPC) increased, and other lipid species (monoglyceride (MG), diglyceride (DG), phosphatidylcholine (PC), phosphatidylglycerol (PG), ceramide (CER)) were unchanged following PIM1 induction (Fig. 5A). Despite an overall reduction, we speculated that specific alterations within the TG lipid class could still positively influence prostate cancer cell proliferation and survival following PIM1 induction. We set cut-off parameters for increase (log2FC ≥ 1, *p* ≤ 0.05) and decrease (log2FC ≥ −1, *p* ≤ 0.05) to identify differential levels of lipid species within the TGs. We observed a significant increase in several TG species (TG(15:0/14:0/15:0), TG(16:0/14:0/16:0), TG(15:0/16:0/16:0), TG(18:0/16:0/20:0), and TG(15:0/16:0/24:0)) with doxycycline treatment (50 ng/ml, 24 h) in PC3TripzPIM1 cells (Fig. 5B). We believe that these TG species, especially TG(15:0/14:0/15:0), which showed approximately 10 log2FC increase, could be critical for PIM1-associated regulation of LD accumulation in prostate cancer. Besides elevated TG levels, there must be a sufficient supply of phospholipids for the LD monolayer to support increased accumulation. Therefore, we measured PC, the most prominent lipid on the LD surface [30], and found a drastic increase at log2FC ≥ 2, *p* ≤ 0.05 in numerous lipid species within this class upon PIM1 induction (Fig. 5C). The production of PC is maintained by enzymes of the Kennedy pathway [31] whereas their remodeling requires re-acylation of LPC, which occurs in the Lands pathway [32]. We also observed increased production of total LPC (Fig. 5A) as well in different lipid species within this class (Fig. 5D), indicating that PIM1 upregulation might induce production of certain LPC funneled into Lands pathway for PC remodeling. The order of phospholipid prevalence from high to low reported on LDs in mammalian cells is PC (60%), PE (24%), PI (8%), PS, and other phospholipids in minor amounts [30]. While other investigations have shown the presence of DG instead of phosphatidic acid [33] or SM as a major phospholipid on their LD surface [34]. In connection with the reported literature about phospholipid prevalence on the LD surface, we measured the remaining lipid classes exhibiting a significant decrease (PE, LPE, PS, PI, and SM) or that were unaltered (DG, MG, and CER) following PIM1 induction (Fig. 5A). Differential levels of various species within these lipid classes were noticed following doxycycline treatment (Fig. S5A–G). Collectively, this data highlights the involvement of PIM1 in altering lipid composition in prostate cancer.

### PIM1 kinase promotes cell survival during nutrient stress in prostate cancer

**Fig. 6:**
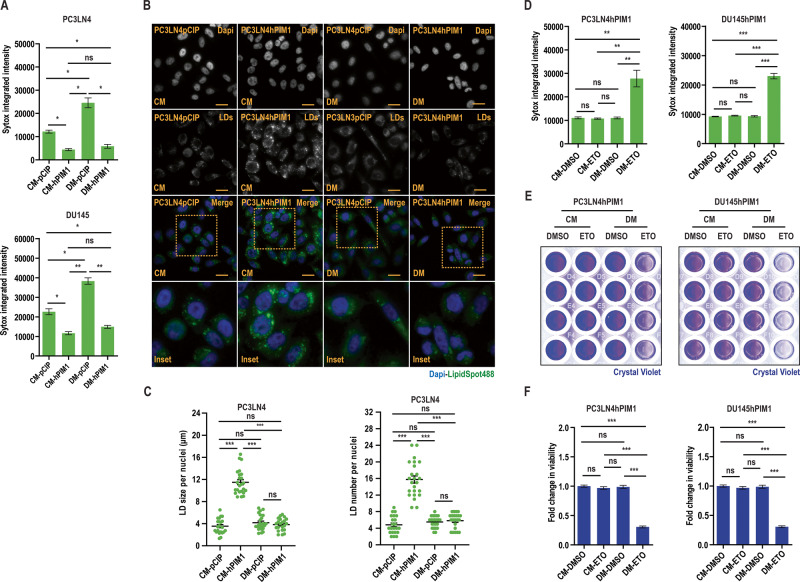
PIM1 promotes cell survival during nutrient stress. **A** Quantitative analysis of SyTox fluorescence intensity indicating relative cell death in the indiated cell lines maintained in complete media (CM, RPMI1640, 10% dialyzed FBS, 25 mM Glucose) or depleted media (DM, RPMI1640, 10% dialyzed FBS) for 24 h. **B** Representative images of LDs in PC3LN4pCIP and PC3LN4hPIM1 cells maintained in complete or depleted media for 48 h. LDs in green (LipidSpot488) and nuclei in blue (Dapi). Scale bars, 50 µm. **C** Quantification of LD size and number per nuclei from (*n* > 30/treatment group). **D** Quantitative analysis of SyTox fluorescence intensity indicating relative cell death in the indicated cell lines maintained in complete or depleted media and treated with DMSO or Etomoxir (ETO, 100 µM, 48 h). **E** Representative images of crystal violet staining of PC3LN4pCIP, PC3LN4hPIM1, DU145pCIP, and DU145hPIM1 cells maintained in complete or depleted media and treated with DMSO or ETO. **F** Quantification of crystal violet staining. *n* = 3, mean ± SEM. *p ≤ 0.05, **p ≤ 0.01, ***p ≤ 0.001.

Solid tumor cells are commonly subjected to nutrient stress due to a lack of functional vascularization, which dictates nutrient supply in their microenvironment [11]. We speculated that prostate cancer cells might accumulate and utilize LDs following PIM1 induction to promote survival under nutrient stress. To test this, we cultured prostate cancer cells (PC3LN4 or DU145) expressing vector (pCIP) or PIM1 (hPIM1) in complete media (CM, RPMI1640, 10% dialyzed FBS, 25 mM Glucose) or introduced nutrient stress by restricting access to glucose (depleted media, DM, RPMI1640, 10% dialyzed FBS) for 24 h, and relative cell death was measured using SyTox, a cell-permeable dye that penetrates compromised plasma membranes characteristic of dead cells. Glucose depletion caused significant cell death in parental DU145 and PC3LN4 cells, whereas little death was observed in cells overexpressing PIM1 (Fig. 6A). Furthermore, the pro-survival effect of PIM1 on prostate cancer cells under nutrient stress was visible within 4 h and maintained through 71 h (Fig. S6A–E, S6H-K). Notably, the increase in LD accumulation, size, or number per nuclei seen with PIM1 upregulation in complete media was reversed following glucose depletion (Fig. 6B, C), alongside increased PIM1 protein levels (Fig. S6F, L) suggesting that these LDs are utilized during nutrient stress to promote survival. While glucose depletion is physiologically relevant to what cancer cells can experience during tumor outgrowth, tumors experience many forms of nutrient stress. To confirm that LD utilization was not specific to glucose depletion, we also treated our WT, TKO, and TKO + PIM1 MEFs with HBSS and monitored LDs over time. We observed a similar reduction in LD size and number following HBSS incubation compared to glucose deprivation, and LD depletion was more complete in cells lacking PIM. (Fig. S6M−R). Next, we investigated whether fatty acid β-oxidation is responsible for the LD-associated survival phenotype exhibited by PIM1 during nutrient stress. To this end, we blocked the mitochondrial fatty acid uptake using carnitine palmitoyltransferase 1 (CPT1) inhibitor (Etomoxir (ETO), 100 µM, 48 h) in PIM1 overexpressing (PC3LN4hPIM1 or DU145hPIM1) cells with or without nutrient stress and measured relative cell death using SyTox. ETO treatment did not induce cell death under normal conditions. In contrast, it dramatically reduced viability during nutrient stress, regardless of PIM1 overexpression (Fig. 6D). This data indicates that blocking the utilization of LD fatty acids during nutrient stress negates the pro-survival effect of PIM1 under nutrient deprivation. Parallel experiments to measure survival with a crystal violet cell viability assay showed similar results (Fig. 6E, F). Taken together, the data show that PIM1 promotes survival during nutrient stress through LD fatty acid utilization in prostate cancer.

### Targeting PIM inhibits LD accumulation and tumor growth in vivo

**Fig. 7:**
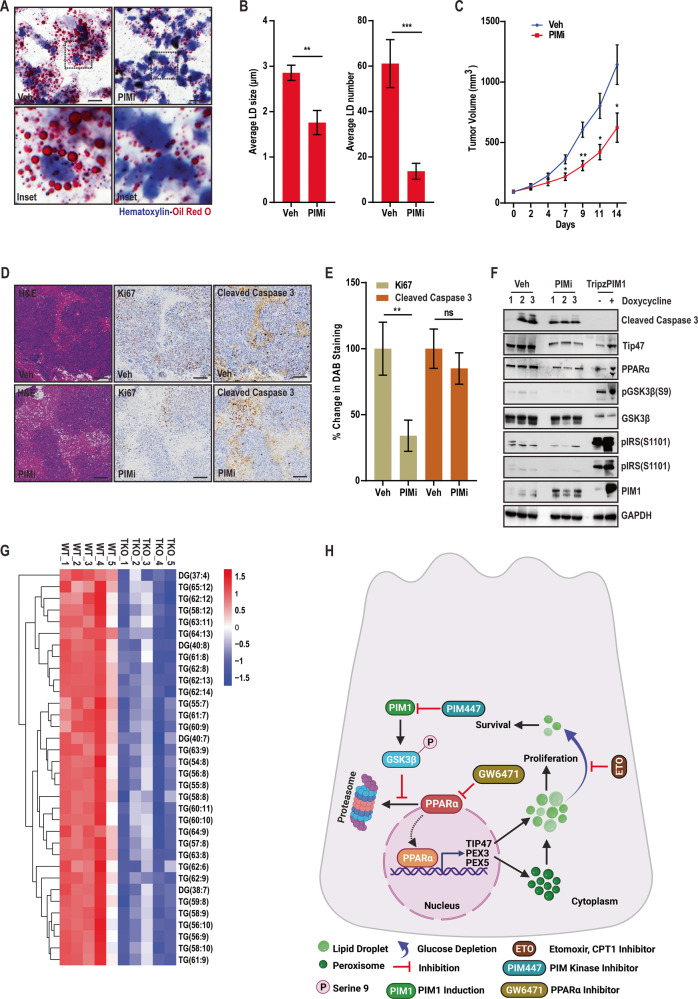
PIM inhibition reduces LD accumulation and tumor growth in vivo. **A** Representative images of LDs in PC3LN4 xenograft tumors following indicated treatment. LDs are in red (Oil Red O) and nuclei in blue (Hematoxylin). Scale bars, 50 µm. **B** Quantification of average LD size and number. *n* = 3, mean ± SEM. **p ≤ 0.01, ***p ≤ 0.001. **C** Tumor volume (mm^3^) determined over time. *n* = 8, mean ± SEM. *p ≤ 0.05, **p ≤ 0.01. **D** Representative images of tumor sections (4 micrometers) examined by H&E and immunohistochemical analysis. Scale bars, 50 µm. **E** Quantification of immunohistochemistry. **F** Western blot analysis of tumor samples alongside indicated cells exhibiting similar patterns in nitro and in vivo following PIM inhibition. **G** Heat map showing downregulation of DG and TG levels in serum samples isolated from indicated mice. **H** working model describing the role of PIM1 kinase in regulating LD accumulation through GSK3β-PPARα axis to promote prostate cancer survival and proliferation.

Based on our findings in vitro, we next investigated the effect of targeting PIM kinase on LD accumulation, tumor growth, and proliferation in vivo. Two million PC3LN4 cells in DPBS were injected subcutaneously into each flank of SCID mice. Once average tumor size reached ~100 mm^3^, mice were randomized for treatment with vehicle (Cremophore EL/Ethanol/DPBS-24/6/70 ratio, p.o. daily) and PIM inhibitor, AZD1208 (30 mg/kg p.o. daily). The effect PIM inhibition on LD accumulation was significant as monitored through oil red o staining (Fig. 7A) and quantified by average size or number (Fig. 7B). Interestingly, the reduction in LD accumulation correlated with retarded tumor growth in PIMi-treated mice evidently at days 7–14 as compared to vehicle (Fig. 7C). Because AZD1208 is no longer a clinical candidate due to toxicity in clinical trials, we also verified these results in PC3 xenograft tumor sections previously isolated from mice treated with PIM447 [35], a PIM inhibitor that has an improved safety profile and is currently being tested in Phase II/III clinical trials. LD staining confirmed that PIM447 significantly reduced LD size and number compared to controls (Fig. S7). In support of our in vitro data showing that targeting PIM1 decreases LD accumulation and cell proliferation, H&E and immunohistochemical analysis showed that PIMi treated tumors tend to be less proliferative (Fig. 7D, E). To confirm the GSK3β-PPARα signaling axis downstream of PIM kinase effectively alters LD accumulation in vivo, we assessed tumor tissues (*n* = 3 tumors/group) by western blotting alongside cell lysates exhibiting PIM1 induction (Fig. 7F). Treatment with PIM inhibitor decreased GSK3β inhibition through ser9 phosphorylation along with PPARα and Tip47 expression (Fig. 7F) linking PIM1 LD accumulation though GSK3β-PPARα signaling axis. Finally, we confirmed the impact of targeting PIM kinase on lipid metabolites in serum collected from WT and TKO mice. To this end, the results revealed disrupted circulation of lipid species (Fig. 7S). In particular, triacylglycerol and diacylglycerols were substantially lower in TKO mice (Fig. 7G). Since triglycerides are one of the main components of LD core, any disruption in their circulation could severely affect their accumulation. These findings indicate that targeting PIM kinase inhibits tumor growth or proliferation by reducing LD accumulation in vivo.

## Discussion

Growing evidence indicates an important role for LD in prostate cancer progression and survival [16, 36–39]. While PIM1 is implicated as an important factor mediating prostate cancer growth and resistance to therapy [20, 40–42], few studies have investigated a potential role for PIM in LD accumulation [23, 43]. Here, we identify PIM1 as a driver of LD accumulation and show a significant effect of PIM1 expression on prostate cancer proliferation and survival, particularly during nutrient stress. Our data indicate that PIM1 acts through a novel signaling axis (GSK3β-PPARα) that involves direct phosphorylation and inhibition of GSK3β and subsequent activation of PPARα. Ultimately, this pathway promotes peroxisomal biogenesis alongside altered lipid composition to facilitate LD accumulation. Moreover, targeting PIM1 or PPARα signaling using genetic or chemical means abrogates the proliferative and survival advantage associated with LD accumulation and PIM1 induction in prostate cancer.

During prostate cancer progression, tumor cells undergo metabolic reprogramming of lipids [44]. Our data show that PIM1 induction alters lipid composition in prostate cancer cells (Fig. 5, S5). Because PIM1-associated lipidomic alterations have not been investigated in solid tumors, we took an unbiased lipidomic approach to define specific changes within each lipid class following PIM1 overexpression, particularly those related to LD formation and accumulation. Esterified fatty acids are incorporated into the LD core and stored as TGs and SEs [45]. Multiple reports suggest that the composition of LDs differs between cancer types and depends on the tumor microenvironment [46, 47]. We observed that PIM1 induction increased the production of specific TG species (Fig. 5B) that are known to facilitate LD accumulation in prostate cancer cells. Interestingly, PIM1 did not affect SEs in our study, indicating its specificity for regulating TGs. Further studies are needed to dissect the precise mechanisms underlying TG accumulation and utilization following PIM1 induction in prostate cancer. A phospholipid monolayer surrounds the neutral lipid core of LDs. We speculate that PIM1-associated changes in specific lipid species in the remaining lipid classes (Fig. 5C, D, S5A-G) fuel LD membrane need during LD accumulation. Recent studies reported that dysregulated PC metabolism is associated with cancer [1, 48–50] and linked key enzymes involved in their metabolism/remodeling to prostate cancer aggressiveness [51]. Interestingly, these enzymes are known to regulate LD membrane synthesis through the production of PCs [30, 31, 52, 53]. Lipidomic analysis revealed an increase in the production of specific PC species (Fig. 5C) required for membrane formation during LD accumulation in PIM1 overexpressing cells. The accumulation of LPC species (Fig. 5C) would ensure optimum PC remodeling through the Lands pathway to complement PC production. The contribution of other phospholipid classes (Fig. S5) associated with PIM1 upregulation toward LD monolayer is minimal and usually context-dependent; nonetheless, it is conceivable these changes might have direct or indirect implications in prostate cancer progression.

This work identifies PIM1 as a key player regulating lipid profile of cancer cells both in vitro and in vivo (Fig. 1, S1). Mechanistically, we provide evidence that PIM1 initiates peroxisomal biogenesis and LD accumulation via a new signaling axis involving GSK3β and PPAR-mediated transcription. We found that PIM1 phosphorylates GSK3β at the inhibition Ser9 site, and this pathway positively correlates with LD accumulation as well as increased LD size or number (Fig. S2B−D). Expression of a non-phosphorylatable mutant of GSK3β (S9A) blocked the ability of PIM1 to increase LDs (Fig. 2D−F), indicating that PIM1-mediated phosphorylation and inhibition of GSK3β relieves a repressive effect on LD accumulation. GSK3 inhibition, either directly (CHIR treatment) or through PIM1 resulted in significant LD accumulation. In addition, targeting GSK3β alone or together with PIM1 has a similar outcome (Fig. S4), suggesting a central role of GSK3β inhibition in regulating peroxisomal biogenesis and LD accumulation in prostate cancer (Figs. 3C−H, S4). Based on these findings, we proposed a model whereby PIM1 drives LD accumulation by phosphorylating and inhibiting GSK3β in prostate cancer (Fig. 7). Thus, identifying a signaling target linked to lipid metabolism downstream of GSK3β inhibition could be insightful for uncovering the mechanism by which PIM1 mediates LD accumulation in prostate cancer.

Alterations in fatty acid availability significantly impact LD accumulation because they undergo esterification to produce neutral lipids that are required for LD formation [4]. Members of the nuclear receptor superfamily, PPARs, are known biological sensors for altered lipid metabolism associated with changes in intracellular fatty acids [27]. Interestingly, GSK3β is known to phosphorylate PPARα at Ser73 and promote its degradation through the ubiquitin-proteasome pathway [27]. Our results show that PIM1 increases nuclear PPARα expression and protein stability through GSK3β inhibition (Fig. S3). PC3-LN4 prostate cancer cells lacking PIM1, which display higher GSK3β activation, show a significant decrease in genes linked to peroxisomal biogenesis (peroxisomal biogenesis factor 3 and 5; PEX3 and PEX5) and LD growth (Tip47) (Fig. 3A, B). Recent studies have highlighted the importance of PPARα modulators for cancer therapy [54]. However, their role in tumorigenesis remains controversial because of conflicting data. On one hand, blocking PPARα inhibits the proliferation of breast [55] and ampullary [56] cancer. On the other hand, PPARα activation showed inhibitory effects on the proliferation of gliomas and ovarian, lung, and colon cancer cells [57]. In the present study, upregulation of PPARα signaling contributed to LD accumulation following PIM1 induction (Fig. 3), which may promote proliferation of prostate cancer cells. Moreover, blocking PPARα signaling using a specific inhibitor, GW6471, was sufficient to reduce LD accumulation and abrogate the effects of PIM1 induction prostate cancer proliferation (Fig. 4).

Restricted access to nutrients has repercussions on both normal and pathological physiology. Upon malignant transformation, cells are more likely to experience stress because nutrient supply in the tumor microenvironment is often compromised due to poor vascularization [11, 58]. LDs regulate cellular metabolism during stress through efficient storage and utilization of lipids [13], which is crucial for restoring energy and redox homeostasis. The energy production during nutrient stress caused by glucose deprivation is primarily dependent on mitochondrial fatty acid oxidation [13]. Interestingly, LDs are known to interact with mitochondria and other cellular organelles, most notably peroxisomes [4, 59]. Peroxisomes are involved in processes that can impact both the biogenesis and breakdown of LDs. As a result, these organelles work in a coordinated fashion to control lipid metabolic flux [60–62]. During periods of low energy, very long chain fatty acids stored as triglycerides in LDs can be transported to peroxisomes to initiate fatty acid oxidation. Our results show that PIM1 increases both peroxisomes and LDs, which can complement each other to provide a survival advantage during nutrient stress caused by glucose depletion (Fig. 6). A possible explanation for this observation is that PIM1 overexpressing prostate cancer cells can effectively deliver fatty acids from the LDs to peroxisomes and mitochondria for energy production to promote survival during nutrient stress. We determine that blocking mitochondrial fatty acid oxidation using etomoxir, a CPT-1 inhibitor, diminishes the survival advantage offered by PIM1 induction or LD accumulation during nutrient stress. Autophagy is shown to be a key defense mechanism during nutrient stress [63, 64], and it has been shown to promote tumor growth by facilitating triglyceride/LD utilization [65]. Therefore, the decreased LD staining observed in PIM1 overexpressing cells during glucose depletion (Fig. 6B) could be attributed to autophagic hydrolysis of LDs releasing fatty acids which then are utilized to restore energy homeostasis and promote cell survival through mitochondrial fatty acid oxidation. Thus, identifying strategies to prevent the utilization of LDs or fatty acids released from LDs represents a promising target for prostate cancer. PIM1 induction is a well-established mechanism causing chemotherapeutic resistance in different tumors, including prostate cancer [66, 67]. We speculate that the utilization of LDs contributes to PIM1-associated resistance to chemotherapy via promoting tumor survival. Thus, further studies are warranted to target PIM1 and/or block LD utilization to increase prostate cancer sensitivity to treatments and overcome resistance.

## Materials and methods

### Cell lines

Parental and genetically modified PC3, PC3LN4, DU145, MEFs, RKO, 293, BEAS-2B, and H1993 cells were cultured in RPMI1640 media supplemented with 10% Fetal Bovine Serum (FBS-Gibco, A5670701) and 1% Penicillin-Streptomycin (Gibco, 15070063) in a humidified atmosphere of 5% CO_2_/95% air at 37 °C. For glucose depletion experiments, indicated cells were washed once with 1x DPBS and then placed into fresh RPMI1640 medium (Gibco, 11879-020) containing 10% dialyzed FBS (Gibco, A33820-01) with or without 25 mM glucose (Thermo Scientific, A24940-01). All cell lines were authenticated by short tandem repeat DNA profiling performed by the University of Arizona Genetics Core facility-Arizona Research Laboratories Division of Biotechnology at the University of Arizona. (http://uagc.arl.arizona.edu/). The cell lines were used for less than 50 passages, and they were routinely tested for mycoplasma contamination.

### Plasmids

pCIP and hPIM1 constructs were created by subcloning into the expression vector pCIG3 (pCMV-IRES-GFP, a gift from Dr. Felicia Goodrum, Addgene, plasmid #78264), modified to replace the GFP cassette with a puromycin resistance gene. TripzEV, TripzPIM1 and HA-PIM1 were gifts from Dr. Andrew S Kraft (UA). hPMI1 (short) was cloned into pCIP (lentiviral backbone) for generation of the hPIM1 cell lines, and PIM1 was cloned into FUCRW (RFP-lentiviral backbone) for generation of RFP-PIM1 cell lines.

### Chemicals

The following chemicals were used at the indicated concentrations: 20, 50, and 100 ng/ml Doxycycline (Sigma-Aldrich, D5207), 50 nM CHIR (Selleckchem, S2745), 3 µM PIM447 (Selleckchem, S7985), 4 µM GW6471 (Selleckchem, S2798), 100 µM Etomoxir (Selleckchem, S8244), 20 µg/ml cycloheximide (Selleckchem, S7418), 1:1000 LipidSpot488 (Biotium, 70065) or LipidSpot610 (Biotium, 70069), 167 nM SyTOX Green Nucleic Acid Stain (Fisher Scientific, S7020), 25 mM Glucose (Thermo Scientific, A24940-01), 10% Dialyzed FBS (Gibco, A33820-01).

### Western blotting

Cultured cells were lysed in RIPA buffer containing protease inhibitors. Protein concentration was determined by Bradford Protein Assay (Bioworld, 20830000-1). Equal protein per well was loaded and resolved by SDS-PAGE. Gels were transferred onto PVDF membranes (Thermo Scientific, 88518) and immunoblotted using the following antibodies: anti-PIM1 (Cell Signaling Technology-CST, 2907), anti-PIM2 (CST, 4730), anti-PIM3 (CST, 4165), anti-phospho IRS-1 (Ser1101-CST, 2385), anti-phospho GSK3β (Ser9-CST, 9323), anti- GSK3β (CST, 12456), anti-GAPDH (CST, 2118), anti-vinculin (CST, 13901), anti-HA-TAG (CST, 3924), anti-cyclin D1 (CST, 2922), anti-β-catenin (Santa Cruz Technology, SC-7963), anti-Tip47 (Santa Cruz Technology, SC-390981), anti-PPARα (LifeSpan BioSciences, LS-B46), anti-βactin (BD Transduction Laboratories, 612656). Anti-rabbit (CST, 7074) and Sheep a-mouse (Prometheus, 84–843) HRP secondary antibodies were used for detection. Blots were imaged on a ChemiDoc (SynGene) using chemiluminescence detection with ECL western blotting substrate (Thermo Scientific, 34095).

### Immunofluorescence

Cells were plated in six-well plates containing microscope coverslips and treated as indicated. After treatment, cells were fixed with 4% formaldehyde for 20 min. and kept in a blocking solution (5% NGS and 0.3% Triton X-100 in PBS) for 60 min. Then, cells were incubated with anti-Catalase (CST, 12980), anti-PPARα (LifeSpan BioSciences, LS-B46), anti-Tom20 (CST, 42406) antibodies for 60 min. Following primary antibody incubation, cells were washed with 1X DPBS and incubated in secondary antibodies (Alexa Fluor 568 goat anti-mouse and Alexa Fluor 488 goat anti-rabbit, 1:500 dilution) for 60 min. Finally, cells were mounted on glass slides with mounting media (CST, Prolong® Gold, 8961) containing DAPI. Images were taken at 60× magnification using a fluorescent microscope. For LD imaging, cells were seeded and fixed in 4% formaldehyde as described above followed by 30 min incubation in 1:1000 diluted LipidSpot 488 or LipidSpot610 (Biotium). After staining, cells were washed in 1X DPBS and mounted onto slides.

### Oil Red O Staining

Oil Red O staining was performed according to the manufacturer’s procedure (Lipid (Oil Red O) staining kit, Sigma-Aldrich, MAK194). Briefly, a stock oil red solution was prepared to dilute 0.7 g Oil Red O with 200 mL isopropanol. A working dilution was then obtained by mixing 6 parts Oil-Red O stock with 4 parts dH2O. Specimens (cells or tissue sections) were first fixed in 10% formalin for 30 min. and incubated in 60% isopropanol for 5 min. Then, specimens were covered in a working oil red solution for 20 min. and kept in hematoxylin for 1 min. At the end of each step, specimens were washed 2–5 times with dH2O. Finally, analyzed immediately by light microscopy.

### Transfection

For transient transfections, cells were seeded at 60% confluency onto glass coverslips placed on 6 well plates. After 24 h, cells were transfected with the indicated constructs using Lipofectamine 3000 transfection reagent (Invitrogen, L3000008), according to the manufacturer’s protocol.

### qRT-PCR analysis

Messenger RNA was isolated from cell lysates using the RNeasy Mini Kit (Qiagen, 74104), and cDNA was synthesized from each sample using the RT^2^ first strand synthesis kit (Qiagen, 330401). Changes in gene expression in response to PIM1 loss were measured as follows: qRT-PCR reactions were performed with equal amounts of starting material (1000 ng RNA) using qPCRBIO SyGreen Blue Mix (PCR Biosystems, PB20.15-01), according to the manufacturer’s protocol. Validated primer sets (QuantiTech primer assays; Qiagen) for each of the following genes were purchased to measure gene expression: PPARα, PPARβ, PPARγ, PEX3, PEX5, PEX7, and Tip47. All primers were ordered from IDT. GAPDH was used to normalize.

### Cell viability assay

The cell viability was measured by crystal violet staining to assess proliferation. Briefly, cells were plated in 96-well plates, treated with inhibitors as indicated for 72 h, fixed in 4% formaldehyde, and stained with 0.1% crystal violet. The cells were lysed in a 1% sodium dodecyl sulfate solution, and absorbance was measured using a microplate reader at a wavelength of 595 nm.

### Cell death assay

Indicated cells were seeded at 5000 cells/well in 96-well Corning plates and allowed to adhere overnight. Cells were gently washed twice with 1x DPBS and replaced with complete media (CM, RPMI1640, 10% dialyzed FBS, 25 mM Glucose) or depleted media (DM, RPMI1640, 10% dialyzed FBS) containing SyTOX Green Nucleic Acid Stain (Fisher Scientific, S7020) at a final concentration of 167 nM and indicated inhibitor treatment. Dead cells were quantified by measuring green fluorescence with a fluorescent plate reader at 504/523 nm excitation/emission. For real-time detection of cell death during glucose depletion, cells were seeded or treated as mentioned above. Following that plates were placed in the IncuCyte® live-cell analysis system and allowed to warm to 37 °C for 30 min. Four phase contrast and green fluorescent images per well were captured every 4 h from 4 to 71 h. The data was analyzed using integrated software to detect and quantify green (dead) cells/image.

### Image analysis

ImageJ and Fiji software was used to process and analyze immunofluorescence images. The size and number of LDs or catalase puncta per nuclei were done on ImageJ/Fiji by applying a uniform threshold function to create a mask followed by the Analyze particle function. PPARα nuclear fluorescence was quantified on ImageJ.

### Lipid extraction

Cell pellets were collected from PC3TripzPIM1 cells treated with DMSO or doxycycline (50 ng/ml, 24 h) and stored at −80 °C until used. All samples were thawed on ice and 0.5 mL water, 1.5 mL chloroform/methanol (2:1, v/v) were added. This was followed by a 1 min vortex and centrifuge at 3000 rpm at 4 °C for 10 min. Then the organic layer was transferred to a new sample tube and dried under nitrogen gas. It was then resuspended with 200 µL isopropanol/methanol (v/v = 1:1) and 5 μL internal standard LPC (12:0) (125 μg/mL) was added. Finally, samples were centrifuged at 12,000 rpm at 4 °C for 10 min. The supernatant was collected and processed for LC-MS analysis.

### LC-MS analysis

Samples were separated on a Thermo Ultimate 3000 LC system equipped with a Phenomenex Kinetex C18 column (100 mm × 2.1 mm, 1.7 μm). Solvent A was acetonitrile/water (v/v = 6:4) containing 10 mmol/L ammonium formate. Solvent B was acetonitrile/isopropanol (v/v = 1:9) containing 10 mmol/L ammonium formate. The flow rate was 0.3 mL/min and the column oven was held at 50 °C. 2 μL of each sample was injected. Following this, the column eluent was introduced to a Thermo Q Exactive mass spectrometer. The mass spectrometer was operated in positive ion mode and negative ion mode respectively with full scan MS at 70,000 resolution and data-dependent MS/MS collected from 200 to 1200 m/z at 17,500 resolutions. The electrospray ionization source was maintained at a spray voltage of 3 kV at positive ion mode and −2.8 kV at negative ion mode with sheath gas at 35 and auxiliary gas at 15 (arbitrary units). The inlet of the mass spectrometer was held at 350 °C, and the S-lens were set to 50%. Following alignment and normalization, all the peaks in ESI- are merged and imported into the SIMCA-P software for analysis.

### In vivo studies

Two million PC3LN4 cells in DPBS were injected subcutaneously into each flank of SCID mice. Once average tumor size reached ~100 mm^3^, mice were randomized for treatment with vehicle (Cremophore EL/Ethanol/DPBS-24/6/70 ratio, p.o. daily) and PIM inhibitor, AZD1208 (30 mg/kg p.o. daily). Tumor volume was monitored by caliper measurements. Fourteen days after treatment, animals were sacrificed, and tumors were harvested. Tumors were fixed, embedded in paraffin, and sectioned for staining with hematoxylin and eosin or antibodies specific for Ki67and CC3. A decent amount of tumor tissue from each group was snap frozen to generate frozen tissue sections for lipid staining. Percent positive staining for the above-mentioned proteins was calculated using ImageJ analysis software. Investigators were blinded to the sample information prior to software-based analysis. Blood was drawn from wild type (WT) and PIM kinase triple knockout (TKO) mice and serum was outsourced to panomebio (Saint Louis, MO, USA) for metabolomic analysis. All animal studies were approved by the Institutional Animal Care and Use Committee at the University of Arizona.

### Statistical analysis

Statistical analysis and graphical representation of the data were performed in GraphPad Prism. Quantification of proliferation, death, LD or catalase size and number, nuclear fluorescence, and lipid classes or species were analyzed using the two-tailed *t*-test as well as by ANOVA unless stated otherwise in figure legends. Statistical significance is denoted in figures as *p ≤ 0.05, **p ≤ 0.01, and ***p ≤ 0.001. Biological replicates are denoted as *n* values and are listed in the figure legends for each experiment. n values represent the number of times an experiment was performed. Data are presented as mean ± SEM.

### Supplementary information

supplemental figs

## Data Availability

All data generated or analyzed during this study are included in this published article and supplementary files. The analysis code and additional files can be found on GitHub: https://github.com/vizzerra/SI-invivostudies; https://github.com/vizzerra/SI-lipidomicanalysis.
