# Supplementary material for: PIM1 drives lipid droplet accumulation to promote proliferation and survival in prostate cancer
Source: Oncogene. 2023 Dec 14;43(6):406–19. doi: 10.1038/s41388-023-02914-0 (PMC10837079; doi:10.1038/s41388-023-02914-0)
Supplement: Supplementary file 1 — supplemental figs [file 41388_2023_2914_MOESM1_ESM.pdf]

PIM1 drives lipid droplet accumulation to promote proliferation and survival in prostate cancer

Shailender S. Chauhan[1,*], Andrea L. Casillas[2], Andres D. Vizzerra[1], Hope Liou[2], Amber N. Clements[2], Caitlyn E. Flores[2], Christopher T. Prevost[3], David F. Kashatus[3], Ashley J. Snider[4], Justin M. Snider[4], and Noel A. Warfel[1,5,*]

[1]Department of Cellular and Molecular Medicine, University of Arizona, Tucson, AZ 85724, USA; [2]Cancer Biology Graduate Program, University of Arizona, Tucson, AZ 85721, USA; [3]Department of Microbiology, Immunology and Cancer Biology, University of Virginia Health System, Charlottesville, VA 22908, USA; [4]Department of Nutritional Sciences, College of Agriculture and Life Sciences, University of Arizona, Tucson, AZ 85721, USA; [5]University of Arizona Cancer Center, University of Arizona, Tucson, AZ 85724, USA.

The file includes:

Supplemental Methods for *in vivo* studies

Fig. S1

Fig. S2

Fig. S3

Fig. S4

Fig. S5

Fig. S6

Fig. S7

Supplemental Methods for *in vivo* studies

Lipid metabolite analysis

Detected metabolomics data were concatenated, and missing values were imputed by using half of the minimum detected intensity for each metabolite. These were log2 transformed prior to statistical analysis. Hypothesis testing was performed with a two-tailed, two-sample t-test with unequal variance. Log2 normalized metabolite intensities were used for null hypothesis testing. The resulting p-values were Benjamini-Hochberg corrected and fold-changed were computed from non-log2 intensities. Overrepresentation analysis was carried out using a Fisher's Exact test. Corrected p-value cutoff of 0.05 and an absolute log2 fold change of 1.0 was used. Significant results (Fig. 7G) were then visualized using the "pheatmap" package in R.

Immunohistochemical analysis

Images taken at 10X magnification were exported as TIFF files in Fiji [1], and quantified by using Color Deconvolution package and H DAB vector option following Crowe and Yue protocol [2]. A total of five different images from each group (Fig. 7D) were used to determine an appropriate maximum threshold for inclusion in analysis for cleaved-caspase-3, and Ki67 protein expression. Analysis was then streamlined using a custom macro once an appropriate threshold was determined. Average mean pixel intensity was used as an indirect way to measure staining area.

Oil Red O staining

Oil Red O staining indicating LDs in tumor samples (Fig. 7A) were quantified by the Color Deconvolution package in Fiji, using "RGB" as the vector option and selecting the red channel for analysis. The maximum threshold was determined using five different images from each group with the goal of excluding non-specific masking, while still retaining individual droplets. This was then streamlined using a custom macro in Fiji.

Scripts availability

https://github.com/vizzerra/SI-invivostudies
https://github.com/vizzerra/SI-lipidomicanalysis

Fig S1

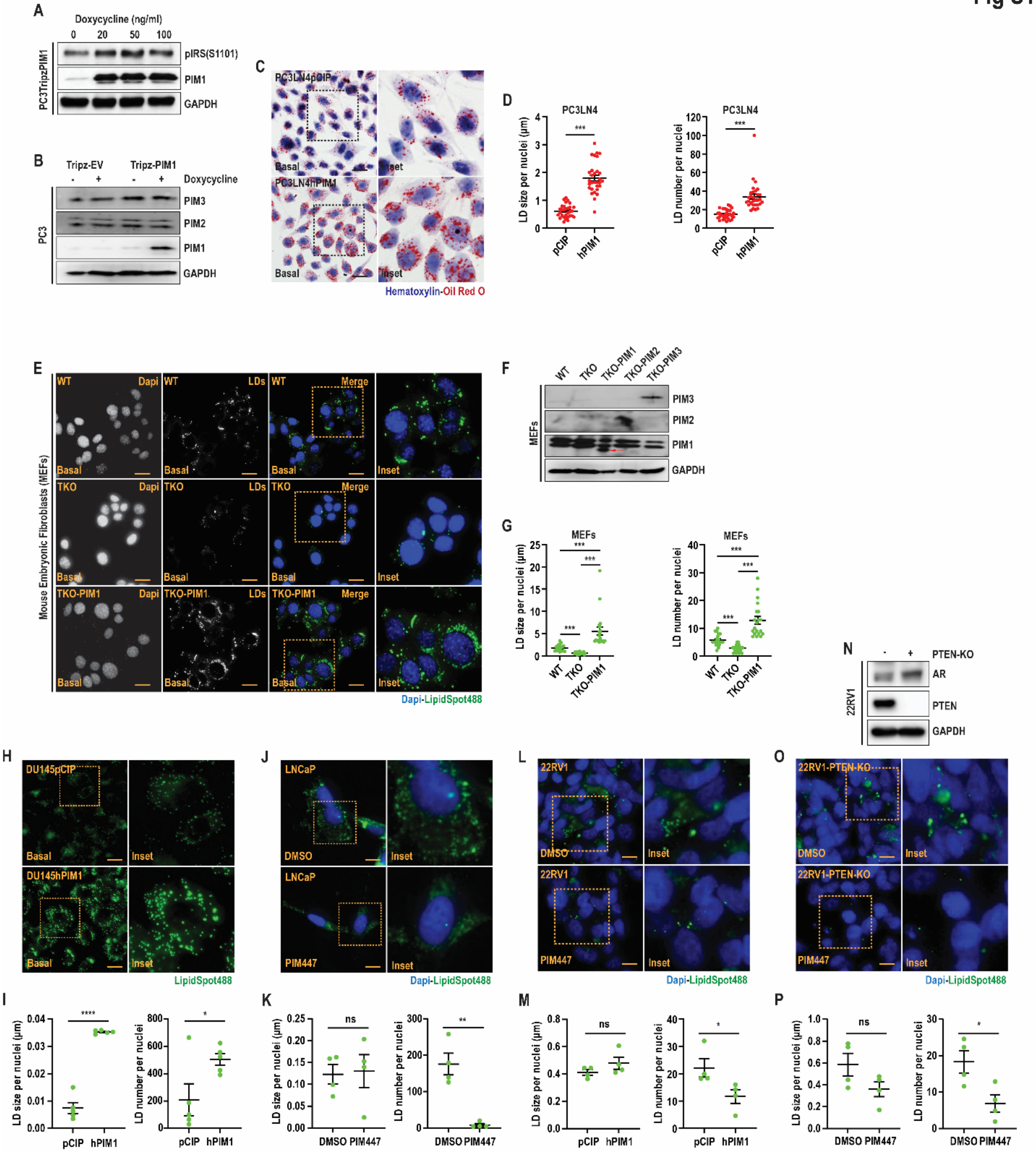

Fig. S1. (A) Western blot confirming increased PIM1 expression and activity in PC3TripzPIM1 cells following doxycycline treatment (24h). (B) Western blot confirming the selectivity of doxycycline-inducible PIM1 (Tripz-PIM1) and empty vector (Tripz-EV) system for PIM1 in indicated cells. (C) Representative images of LDs at basal level in PC3LN4pCIP and PC3LN4hPIM1 cells. LDs are in red (Oil Red O) and nuclei in blue (Hematoxylin). Scale bars, 50 μm. (D) Quantification of LD size and number per nuclei from (C). (E) Representative images of LDs at basal level in mouse embryonic fibroblasts (MEFs), WT (expressing wild-type PIM1), TKO (triple knockout, lacking PIM1, PIM2, and PIM3), TKO-PIM1 (overexpressing PIM1). LDs in green

(LipidSpot488) and nuclei in blue (Dapi). Scale bars, 50 µm. (F) Western blot confirming PIM1 expression at basal level in MEFs, WT, TKO, TKO-PIM1, TKO-PIM2 (overexpressing PIM2), and TKO-PIM3 (overexpressing PIM3). (G) Quantification of LD size and number per nuclei from (E). Representative images of LDs at basal level in DU145pCIP and DU145hPIM1 cells (H) and with PIM inhibition (3 µM, 24h) in LNCaP (J), 22RV1 wild type (L), and in 22RV1 PTEN-KO (lacking PTEN; confirmed using western blot analysis (N)) (O). LDs in green (LipidSpot488) and nuclei in blue (Dapi). Scale bars, 50 µm. Quantification of LD size and number per nuclei from H (I), J (K), L (M), and O (P). For quantification (D), (G), (I), (K), (M), and (P) at least 30 cells were analyzed per treatment group. n=3, mean ± SEM. ***$p \leq 0.001$.

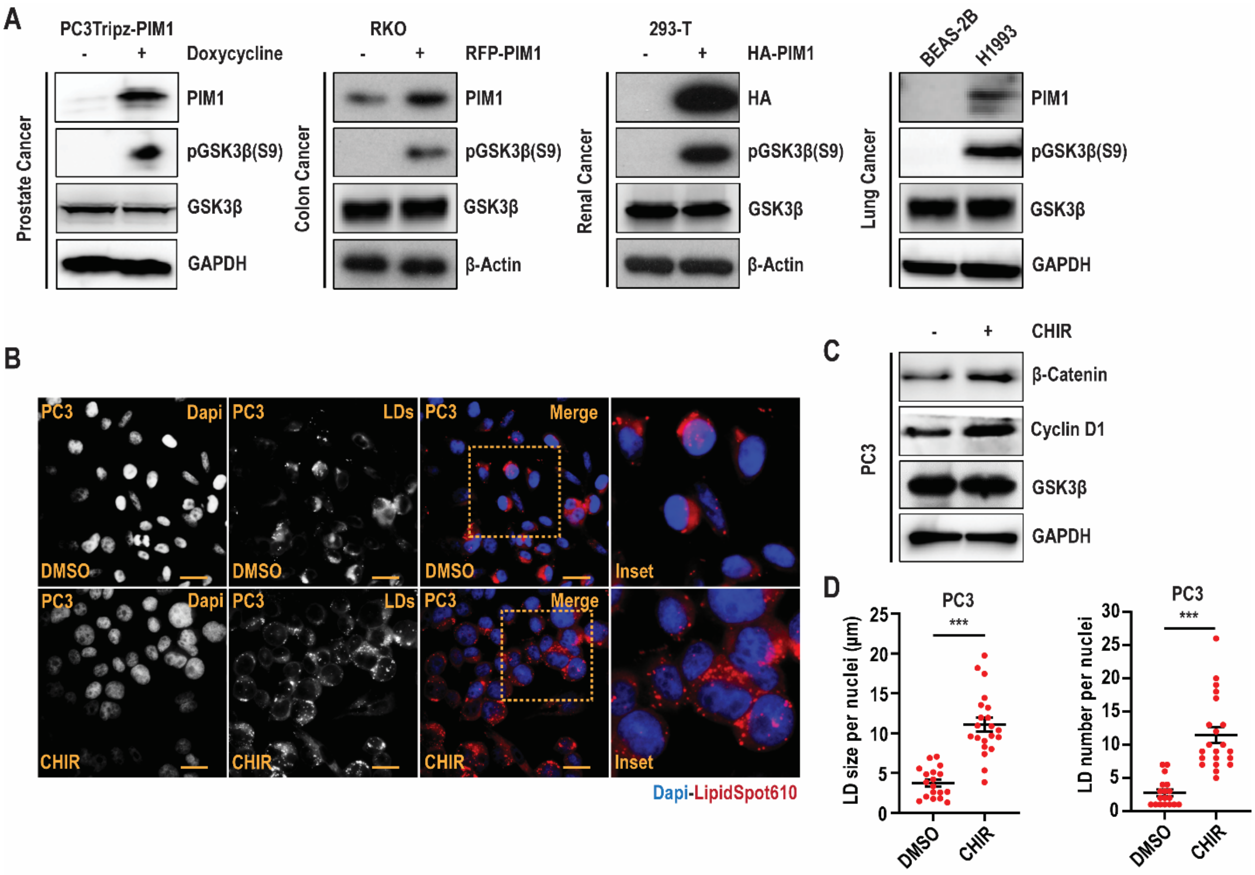

Fig. S2. (A) Western blot confirming the GSK3β inhibition (increased serine 9 (S9) phosphorylation) with PIM1 upregulation in prostate cancer cells (PC3Tripz-PIM1), following doxycycline treatment (50ng/ml, 24h) and in indicated cancer models. (B) Representative images of LDs in PC3 cells following CHIR treatment (50 nM, 24h). LDs in red (LipidSpot610) and nuclei in blue (Dapi). Scale bars, 50 µm. (C) Western blot confirming GSK3β inhibition (increased β-Catenin and Cyclin D1 levels) following CHIR treatment (50 nM, 24h). (D) Quantification of LD size and number per nuclei from (B). For quantification (D), at least 30 cells were analyzed per treatment group. n=3, mean ± SEM. *$p \leq 0.05$, ***$p \leq 0.001$.

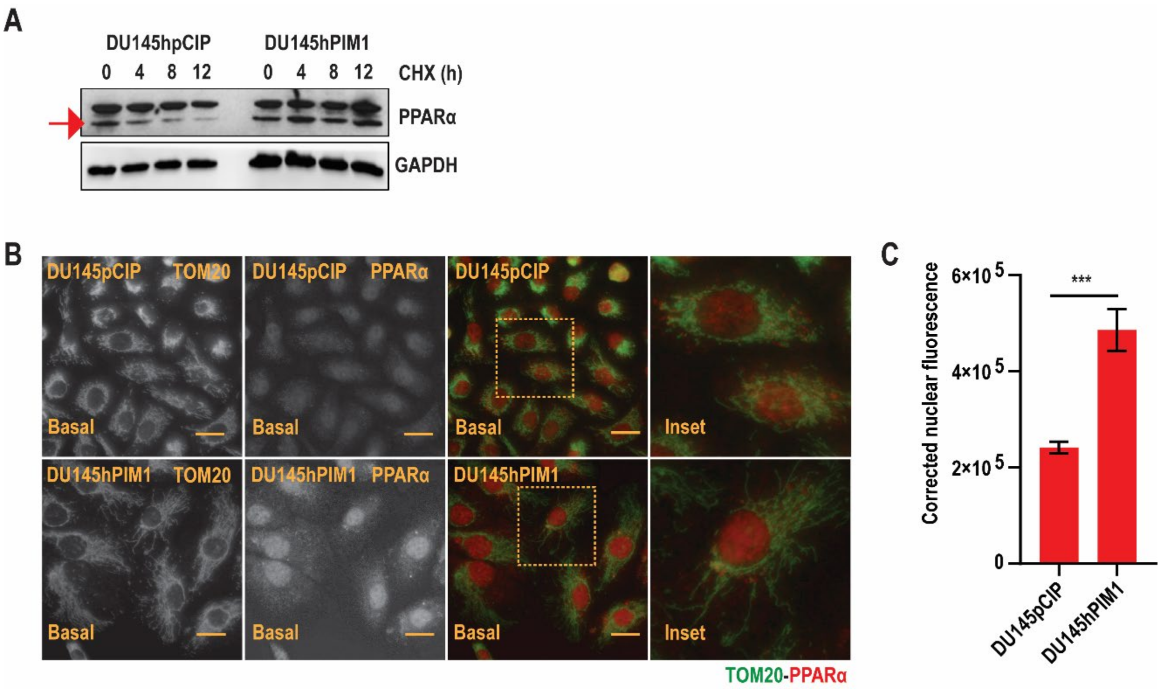

Fig. S3. (A) Western blot confirming PPARα protein stability following cycloheximide (CHX) chase assay in DU145pCIP and DU145hPIM1 cells, PPARα (red arrow). (B) Representative images of PPARα staining at basal in DU145pCIP and DU145hPIM1 cells. PPARα in red and TOM20 in green, Scale bars, 50 μm. (C) Quantification of nuclear PPARα fluorescence intensity from (B). At least 40 cells were analyzed per treatment group. n=3, mean ± SEM. ***$p \leq 0.001$.

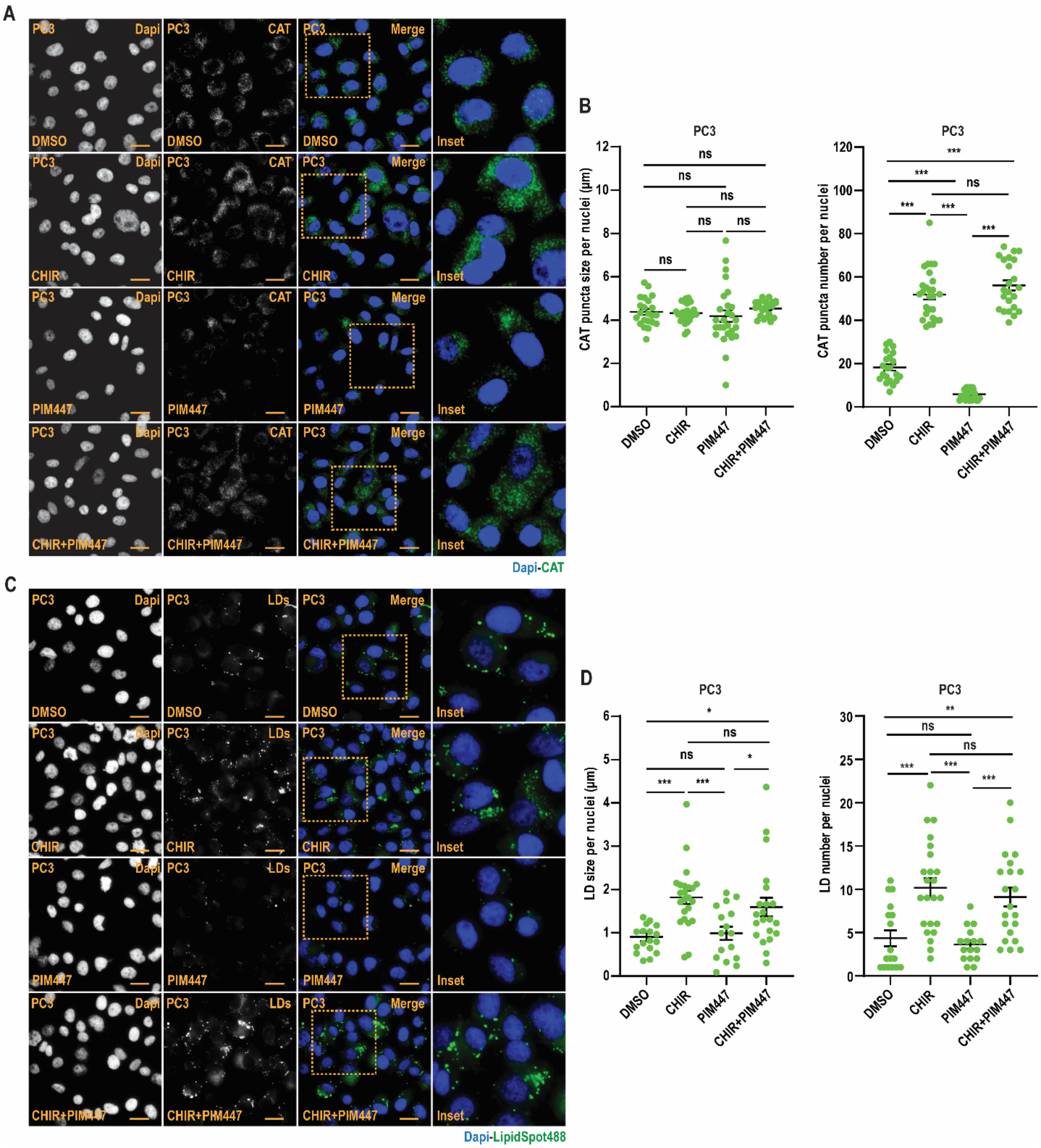

Fig. S4. (A) Representative images of peroxisomal marker catalase (CAT) in PC3 cells following CHIR treatment (50 nM, 24h), alone, and in combination with PIM inhibition (PIM447, 3 µM, 24h). CAT in green, and nuclei in blue (Dapi). Scale bars, 50 µm. (B) Quantification of CAT puncta size and number per nuclei from (A). (C) Representative images of LDs in PC3 cells following CHIR treatment (50 nM, 24h), alone, and in combination with PIM inhibition (PIM447, 3 µM, 24h). LD in green (LipidSpot488), and nuclei in blue (Dapi). Scale bars, 50 µm. (D) Quantification of LD size and number per nuclei from (C). For quantification (B) and (D), at least 30 cells were analyzed per treatment group. n=3, mean ± SEM. *$p \leq 0.05$, **$p \leq 0.01$, and ***$p \leq 0.001$.

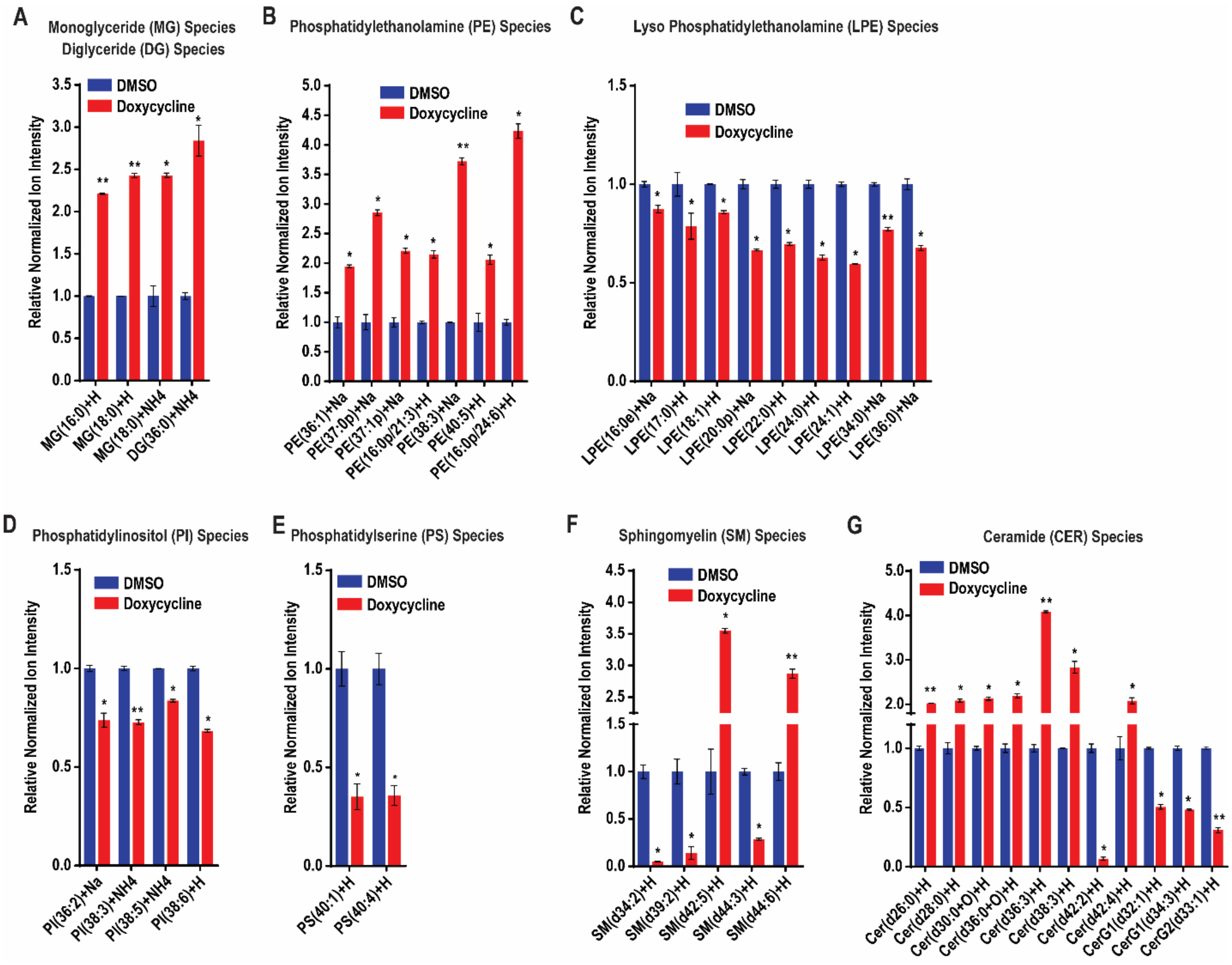

Fig. S5. (A-H) Relative normalized ion intensity values detected (A) monoglyceride and diglyceride, (B) phosphatidylcholine, (C), lyso phosphatidylethanolamine, (D) phosphatidylinositol, (E) phosphatidylserine, (F) sphingomyelin, and (G) ceramide. PC3Tripz-PIM1 cells were treated with DMSO or doxycycline (50ng/ml, 24h) and lipids were extracted by modified Folch method and analyzed by LC-MS/MS. Raw values were normalized and fold change vs DMSO was calculated to generate relative normalized ion intensity values. n=3, mean ± SEM. *p ≤ 0.05, **p ≤ 0.01, ***p ≤ 0.001.

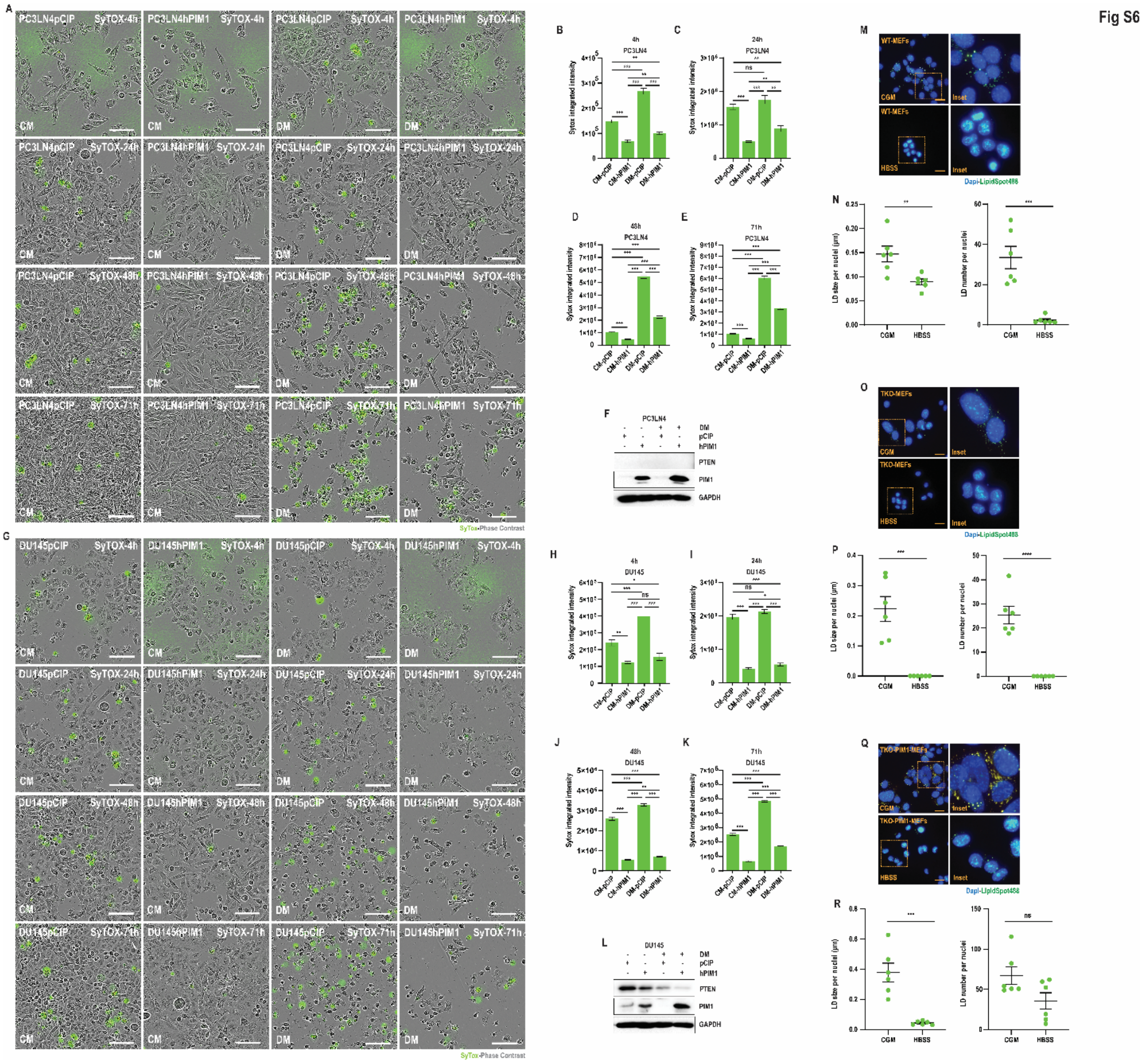

Fig. S6. (A) Representative images of SyTox staining and phase contrast indicating relative cell death in PC3LN4pCIP and PC3LN4hPIM1 cells maintained in complete media (CM, RPMI1640, 10% dialyzed FBS, 25 mM Glucose) or depleted media (DM, RPMI1640, 10% dialyzed FBS) for different time points (4h, 24h, 48h, and 71h). SyTox in green and phase contrast in grey). Scale bars, 100 µm. (B-E) Quantification of SyTox fluorescence intensity for indicated time points from (A). n=3, mean ± SEM. *p ≤ 0.05, **p ≤ 0.01, ***p ≤ 0.001. (F) Western blot showing PIM1 expression in indicated cells maintained in complete media (CM, RPMI1640, 10% dialyzed FBS, 25 mM Glucose) or depleted media (DM, RPMI1640, 10% dialyzed FBS). (G) Representative images of SyTox staining and phase contrast indicating relative cell death in PC3LN4pCIP and PC3LN4hPIM1 cells maintained in complete media (CM, RPMI1640, 10% dialyzed FBS, 25 mM Glucose) or depleted media (DM, RPMI1640, 10% dialyzed FBS) for different time points (4h, 24h, 48h, and 71h). SyTox in green and phase contrast in grey). Scale bars, 100 µm. (H-K) Quantification of SyTox fluorescence intensity for indicated time points from (A). n=3, mean ± SEM. *p ≤ 0.05, **p ≤ 0.01, ***p ≤ 0.001. (L) Western blot showing PIM1 expression in indicated

cells maintained in complete media (CM, RPMI1640, 10% dialyzed FBS, 25 mM Glucose) or depleted media (DM, RPMI1640, 10% dialyzed FBS). Representative images of LDs following HBSS (24h) or complete growth media (CGM) incubation in mouse embryonic fibroblasts (MEFs), WT (expressing wild-type PIM1) (M), TKO (triple knockout, lacking PIM1, PIM2, and PIM3) (O), TKO-PIM1 (overexpressing PIM1) (Q). LDs in green (LipidSpot488) and nuclei in blue (Dapi). Scale bars, 50 μm. Quantification of LD size and number per nuclei from M (N), O (P), and Q (R). For quantification (N), (P), and (R) at least 30 cells were analyzed per treatment group. n=3, mean ± SEM. ***$p \leq 0.001$.

Fig S7

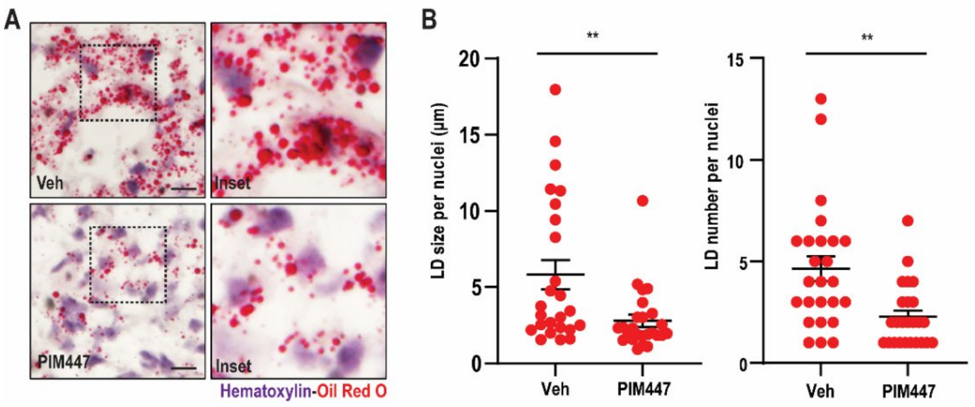

Fig. S7. (A) Representative images of LDs in PC3LN4 xenograft tumors following indicated treatment. LDs are in red (Oil Red O) and nuclei in blue (Hematoxylin). Scale bars, 50 μm. (B) Quantification of average LD size and number. n=3, mean ± SEM. **p ≤ 0.01, ***p ≤ 0.001.

References

1 Schindelin J, Arganda-Carreras I, Frise E, Kaynig V, Longair M, Pietzsch T *et al*. Fiji: an open-source platform for biological-image analysis. *Nat Methods* 2012; 9: 676-682.

2 Crowe AR, Yue W. Semi-quantitative Determination of Protein Expression using Immunohistochemistry Staining and Analysis: An Integrated Protocol. *Bio Protoc* 2019; 9.
